# Quantitative phase imaging with scanning holographic microscopy: an experimental assesment

**DOI:** 10.1186/1475-925X-5-63

**Published:** 2006-11-28

**Authors:** Guy Indebetouw, Yoshitaka Tada, John Leacock

**Affiliations:** 1 Virginia Tech, Physics Department, Blacksburg, VA 24061/0435, USA

## Abstract

This paper demonstrates experimentally how quantitative phase information can be obtained in scanning holographic microscopy. Scanning holography can operate in both coherent and incoherent modes, simultaneously if desired, with different detector geometries. A spatially integrating detector provides an incoherent hologram of the object's intensity distribution (absorption and/or fluorescence, for example), while a point detector in a conjugate plane of the pupil provides a coherent hologram of the object's complex amplitude, from which a quantitative measure of its phase distribution can be extracted. The possibility of capturing simultaneously holograms of three-dimensional specimens, leading to three-dimensional reconstructions with absorption contrast, reflectance contrast, fluorescence contrast, as was previously demonstrated, and quantitative phase contrast, as shown here for the first time, opens up new avenues for multimodal imaging in biological studies.

## 1- Background

Microscopy is an essential tool in biological research, micromechanical testing, the integrated circuit industry, etc. The demand for higher resolution and contrast, shorter acquisition time, and multimodal imaging, among other desirable properties, has resulted in the recent invention, demonstration, and often rapid commercialization of a number of new technologies. In biological studies, two modalities appear to be of primary importance. They are fluorescence imaging for the specific identification of biomolecules in a labeled sample, and phase imaging for the determination of internal structures in unstained specimens. The conventional phase imaging methods (i.e. Zernike phase contrast, and Nomarski differential interference contrast) usually provide only the visualization of the phase of biological structures in a qualitative way, although it is possible to extract quantitative phase information with the differential interference contrast method [1]. Recently, quantitative phase imaging, as provided by digital holographic microscopy [2] gave a new dimension to phase imaging by allowing the quantitative measurement of, for example, biomasses and fluid concentrations in cells. Quantitative phase imaging is also essential in measuring non-destructively the surface topography of biological samples, as well as of micromechanical systems, and integrated electrical circuits.

To our knowledge, no single instrument or imaging method can capture both fluorescence and quantitative phase information of 3D specimens simultaneously in holographic form. In this paper, we demonstrate experimentally that scanning holographic microscopy [3,4], which was developed to obtain holograms of incoherent objects [5,6], and has recently been shown to provide high resolution images of 3D fluorescent biological specimens [7], is also capable of providing quantitative phase information. The possibility of phase imaging by scanning holographic microscopy had been suggested earlier on theoretical grounds [5], but has not yet been demonstrated experimentally. This possibility opens up new potentials for multimodal imaging. For example, it would be possible to obtain, simultaneously if desired, absorption images carrying information of structural arrangements, fluorescence images of labeled specimens revealing functional activities, and quantitative phase images from which internal structures, biomasses, density, etc. could be measured.

The paper is organized as follows. Section two is a brief theoretical review of the two possible operation modes of scanning holography: the incoherent mode giving holograms of the object's intensity distribution (absorption, reflection, and fluorescence), and the coherent mode giving holograms of the object's complex amplitude distribution, and providing a quantitative measure of its phase distribution. Section three describes the experimental set up, and section 4 presents experimental results of phase images of unstained biological specimens, as well as of the quantitative profile of manufactured phase objects. The latter result is compared quantitatively with atomic force microscope measurements. Section five is a brief summary.

## 2- Theory

**Figure 1:**
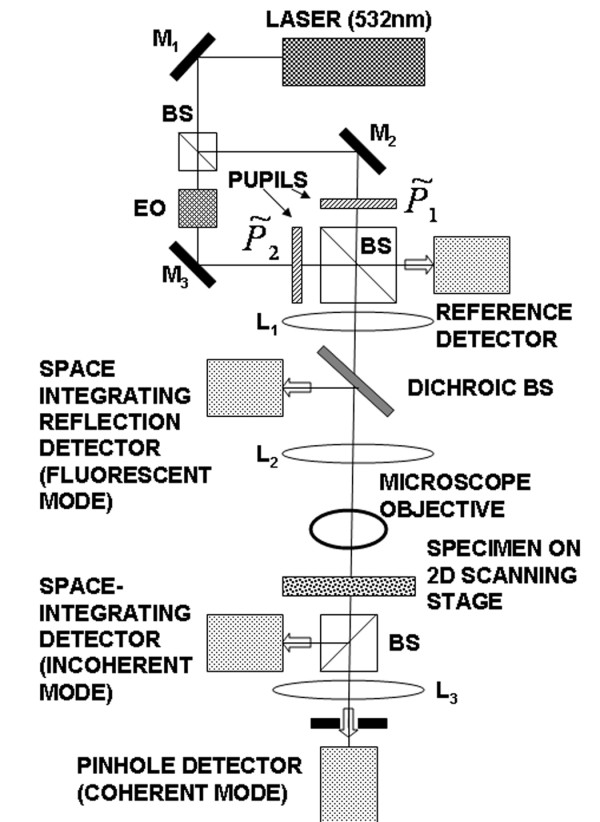
**Experimental set up of a scanning holographic microscope**. Sketch of the experimental setup. M's are mirrors, BS's are beam splitters, EO is an electro-optic phase modulator. Lens L1 (achromat doublet 16 cm focal length) produces the desired Fresnel pattern in the focal plane of L2 (achromat doublet 16 cm focal length). L2 and the objective form a 4-f system projecting a reduced image of the Fresnel pattern onto the specimen. L3 (achromat doublet 12 cm focal length) projects the images of the pupils on the pinhole detector. (Not shown are the beam expanders used to illuminate the pupils).

Scanning holography is a two-pupil interaction method [8] by which incoherent imaging with complex point-spread-functions (PSF) is possible. The method has recently been applied to the recording of high resolution holographic images of incoherent objects and fluorescent biological specimens [6,7]. A single-sideband in-line Fresnel hologram is obtained by a 2D raster scan of the object with the superposed 3D diffraction distributions of two pupils, as sketched in fig. 1. The two pupil distributions P˜1(ρ→)
 MathType@MTEF@5@5@+=feaafiart1ev1aaatCvAUfKttLearuWrP9MDH5MBPbIqV92AaeXatLxBI9gBaebbnrfifHhDYfgasaacH8akY=wiFfYdH8Gipec8Eeeu0xXdbba9frFj0=OqFfea0dXdd9vqai=hGuQ8kuc9pgc9s8qqaq=dirpe0xb9q8qiLsFr0=vr0=vr0dc8meaabaqaciaacaGaaeqabaqabeGadaaakeaacuWGqbaugaacamaaBaaaleaacqaIXaqmaeqaaOGaeiikaGccciGaf8xWdiNbaSaacqGGPaqkaaa@3295@ and P˜2(ρ→)
 MathType@MTEF@5@5@+=feaafiart1ev1aaatCvAUfKttLearuWrP9MDH5MBPbIqV92AaeXatLxBI9gBaebbnrfifHhDYfgasaacH8akY=wiFfYdH8Gipec8Eeeu0xXdbba9frFj0=OqFfea0dXdd9vqai=hGuQ8kuc9pgc9s8qqaq=dirpe0xb9q8qiLsFr0=vr0=vr0dc8meaabaqaciaacaGaaeqabaqabeGadaaakeaacuWGqbaugaacamaaBaaaleaacqaIYaGmaeqaaOGaeiikaGccciGaf8xWdiNbaSaacqGGPaqkaaa@3297@ from the same source (for example, but not necessarily, a laser) are combined by a beam splitter in the pupil plane of the objective where they interfere, forming a Fresnel pattern with a depth-dependent Fresnel number. To obtain the conventional point-spread function of wide field imaging, *P*_1 _is chosen as a point source, and *P*_2 _as a spherical wave with appropriate curvature. The 3D specimen is placed in the focal region of the objective, and scattered lights (transmitted, reflected, and fluorescent) are collected by non-imaging detectors. The hologram data can be obtained by heterodyne detection with one of the pupils shifted in frequency (as done in this work), or by a homodyne method requiring the capture of at least three frames with different relative phases between the two pupils [9]. The amplitude distribution of the illuminating beam, in a transverse plane at an axial distance *z *from the focal plane of the objective, is the Fourier transform of the combined pupil distributions [10]. Namely:

S(r→,z)=F−1{[P˜1(ρ→;z)+P˜2(ρ→;z)exp⁡(−iΩt]},     (1a)
 MathType@MTEF@5@5@+=feaafiart1ev1aaatCvAUfKttLearuWrP9MDH5MBPbIqV92AaeXatLxBI9gBaebbnrfifHhDYfgasaacH8akY=wiFfYdH8Gipec8Eeeu0xXdbba9frFj0=OqFfea0dXdd9vqai=hGuQ8kuc9pgc9s8qqaq=dirpe0xb9q8qiLsFr0=vr0=vr0dc8meaabaqaciaacaGaaeqabaqabeGadaaakeaacqWGtbWucqGGOaakcuWGYbGCgaWcaiabcYcaSiabdQha6jabcMcaPiabg2da9iabdAeagnaaCaaaleqabaGaeyOeI0IaeGymaedaaOWaaiWabeaadaWadaqaaiqbdcfaqzaaiaWaaSbaaSqaaiabigdaXaqabaGccqGGOaakiiGacuWFbpGCgaWcaiabcUda7iabdQha6jabcMcaPiabgUcaRiqbdcfaqzaaiaWaaSbaaSqaaiabikdaYaqabaGccqGGOaakcuWFbpGCgaWcaiabcUda7iabdQha6jabcMcaPiGbcwgaLjabcIha4jabcchaWjabcIcaOiabgkHiTiabdMgaPjabfM6axjabdsha0bGaay5waiaaw2faaaGaay5Eaiaaw2haaiabcYcaSiaaxMaacaWLjaWaaeWaaeaacqaIXaqmcqqGHbqyaiaawIcacaGLPaaaaaa@5D95@

where

P˜1,2(ρ→;z)=P˜1,2(ρ→)exp⁡(iπλzρ2)     (1b)
 MathType@MTEF@5@5@+=feaafiart1ev1aaatCvAUfKttLearuWrP9MDH5MBPbIqV92AaeXatLxBI9gBaebbnrfifHhDYfgasaacH8akY=wiFfYdH8Gipec8Eeeu0xXdbba9frFj0=OqFfea0dXdd9vqai=hGuQ8kuc9pgc9s8qqaq=dirpe0xb9q8qiLsFr0=vr0=vr0dc8meaabaqaciaacaGaaeqabaqabeGadaaakeaacuWGqbaugaacamaaBaaaleaacqaIXaqmcqGGSaalcqaIYaGmaeqaaOGaeiikaGccciGaf8xWdiNbaSaacqGG7aWocqWG6bGEcqGGPaqkcqGH9aqpcuWGqbaugaacamaaBaaaleaacqaIXaqmcqGGSaalcqaIYaGmaeqaaOGaeiikaGIaf8xWdiNbaSaacqGGPaqkcyGGLbqzcqGG4baEcqGGWbaCcqGGOaakcqWGPbqAcqWFapaCcqWF7oaBcqWG6bGEcqWFbpGCdaahaaWcbeqaaiabikdaYaaakiabcMcaPiaaxMaacaWLjaWaaeWaaeaacqaIXaqmcqqGIbGyaiaawIcacaGLPaaaaaa@53A8@

are the generalized defocused pupils [11]. *F*^-1 ^stands for inverse Fourier transform, and Ω is the frequency shift of one of the pupils. The transverse spatial frequency vector ρ→
 MathType@MTEF@5@5@+=feaafiart1ev1aaatCvAUfKttLearuWrP9MDH5MBPbIqV92AaeXatLxBI9gBaebbnrfifHhDYfgasaacH8akY=wiFfYdH8Gipec8Eeeu0xXdbba9frFj0=OqFfea0dXdd9vqai=hGuQ8kuc9pgc9s8qqaq=dirpe0xb9q8qiLsFr0=vr0=vr0dc8meaabaqaciaacaGaaeqabaqabeGadaaakeaaiiGacuWFbpGCgaWcaaaa@2E85@ = r→
 MathType@MTEF@5@5@+=feaafiart1ev1aaatCvAUfKttLearuWrP9MDH5MBPbIqV92AaeXatLxBI9gBaebbnrfifHhDYfgasaacH8akY=wiFfYdH8Gipec8Eeeu0xXdbba9frFj0=OqFfea0dXdd9vqai=hGuQ8kuc9pgc9s8qqaq=dirpe0xb9q8qiLsFr0=vr0=vr0dc8meaabaqaciaacaGaaeqabaqabeGadaaakeaacuWGYbGCgaWcaaaa@2E2B@_*P*_/*λf*_0 _is proportional to the real space coordinate r→
 MathType@MTEF@5@5@+=feaafiart1ev1aaatCvAUfKttLearuWrP9MDH5MBPbIqV92AaeXatLxBI9gBaebbnrfifHhDYfgasaacH8akY=wiFfYdH8Gipec8Eeeu0xXdbba9frFj0=OqFfea0dXdd9vqai=hGuQ8kuc9pgc9s8qqaq=dirpe0xb9q8qiLsFr0=vr0=vr0dc8meaabaqaciaacaGaaeqabaqabeGadaaakeaacuWGYbGCgaWcaaaa@2E2B@_*P *_in the pupil plane [10]. *λ *is the wavelength of the illumination, and *f*_0 _is the focal length of the objective. The two pupils used to obtain an in-line Fresnel hologram are, respectively, a spherical wave filling the pupil of the objective, and a point at the center of that pupil:

P˜1(ρ→)
 MathType@MTEF@5@5@+=feaafiart1ev1aaatCvAUfKttLearuWrP9MDH5MBPbIqV92AaeXatLxBI9gBaebbnrfifHhDYfgasaacH8akY=wiFfYdH8Gipec8Eeeu0xXdbba9frFj0=OqFfea0dXdd9vqai=hGuQ8kuc9pgc9s8qqaq=dirpe0xb9q8qiLsFr0=vr0=vr0dc8meaabaqaciaacaGaaeqabaqabeGadaaakeaacuWGqbaugaacamaaBaaaleaacqaIXaqmaeqaaOGaeiikaGccciGaf8xWdiNbaSaacqGGPaqkaaa@3295@ = exp(*iπλz*_0_*ρ*^2^)*circ*(*ρ*/*ρ*_*MAX*_)

P˜2(ρ→)=δ(ρ→).     (2)
 MathType@MTEF@5@5@+=feaafiart1ev1aaatCvAUfKttLearuWrP9MDH5MBPbIqV92AaeXatLxBI9gBaebbnrfifHhDYfgasaacH8akY=wiFfYdH8Gipec8Eeeu0xXdbba9frFj0=OqFfea0dXdd9vqai=hGuQ8kuc9pgc9s8qqaq=dirpe0xb9q8qiLsFr0=vr0=vr0dc8meaabaqaciaacaGaaeqabaqabeGadaaakeaacuWGqbaugaacamaaBaaaleaacqaIYaGmaeqaaOGaeiikaGccciGaf8xWdiNbaSaacqGGPaqkcqGH9aqpcqWF0oazcqGGOaakcuWFbpGCgaWcaiabcMcaPiabc6caUiaaxMaacaWLjaWaaeWaaeaacqaIYaGmaiaawIcacaGLPaaaaaa@3D5F@

*circ*(*x*) is a disc function of unit radius. *ρ*_*MAX *_= sin*α*/*λ *is the cutoff frequency of the objective, where sin*α *= *NA *is its numerical aperture. The Fresnel pattern projected on the object is the interference of the Fourier transforms of the two pupils, namely a spherical wave and a plane wave in the paraxial approximation. The Fresnel number of this pattern is determined by the free parameter *z*_0_, which is the distance from the objective's focal plane to the point where the spherical wave comes to a focus.

There are two possible modes of operation, depending on the detector geometry [5]. With a spatially integrating detector, the resulting data is a convolution of the object's intensity distribution with the desired complex PSF (namely, a spherical wave with a radius of curvature *z*_0 _+ *z*, and a radius *a *= *z*_0_sin*α*, in this case). This detection mode leads to a hologram from which the three-dimensional distribution of scattering intensity, absorption, and fluorescence intensity can be reconstructed. With a point detector at the center of a conjugate pupil plane, the resulting data is a convolution of the object's complex amplitude distribution with the same complex PSF. The reconstruction of this hologram gives the three-dimensional distribution of the specimen's complex amplitude transmittance. In particular, assuming that multiple scattering can be ignored, the phase of the reconstruction is a quantitative measure of the integrated optical path length through the specimen. These two modes of operation are similar to the usual coherent/incoherent imaging modes of a conventional system, which are obtained by using, respectively, a point source, or a large spatially incoherent source. In scanning holography, the detector size plays a similar role to that of the source size in conventional imaging.

For simplicity, let's assume an object with an amplitude transmittance *T*(r→
 MathType@MTEF@5@5@+=feaafiart1ev1aaatCvAUfKttLearuWrP9MDH5MBPbIqV92AaeXatLxBI9gBaebbnrfifHhDYfgasaacH8akY=wiFfYdH8Gipec8Eeeu0xXdbba9frFj0=OqFfea0dXdd9vqai=hGuQ8kuc9pgc9s8qqaq=dirpe0xb9q8qiLsFr0=vr0=vr0dc8meaabaqaciaacaGaaeqabaqabeGadaaakeaacuWGYbGCgaWcaaaa@2E2B@, *z*). Note that for an incoherent or fluorescent object, the phase of the transmitted field is a random variable, and only the intensity *I*(r→
 MathType@MTEF@5@5@+=feaafiart1ev1aaatCvAUfKttLearuWrP9MDH5MBPbIqV92AaeXatLxBI9gBaebbnrfifHhDYfgasaacH8akY=wiFfYdH8Gipec8Eeeu0xXdbba9frFj0=OqFfea0dXdd9vqai=hGuQ8kuc9pgc9s8qqaq=dirpe0xb9q8qiLsFr0=vr0=vr0dc8meaabaqaciaacaGaaeqabaqabeGadaaakeaacuWGYbGCgaWcaaaa@2E2B@, *z*) = |*T*(r→
 MathType@MTEF@5@5@+=feaafiart1ev1aaatCvAUfKttLearuWrP9MDH5MBPbIqV92AaeXatLxBI9gBaebbnrfifHhDYfgasaacH8akY=wiFfYdH8Gipec8Eeeu0xXdbba9frFj0=OqFfea0dXdd9vqai=hGuQ8kuc9pgc9s8qqaq=dirpe0xb9q8qiLsFr0=vr0=vr0dc8meaabaqaciaacaGaaeqabaqabeGadaaakeaacuWGYbGCgaWcaaaa@2E2B@, *z*)|^2 ^is measurable. For a quasi transparent object, the phase is equal to the integrated optical thickness of the object: Φ(r→
 MathType@MTEF@5@5@+=feaafiart1ev1aaatCvAUfKttLearuWrP9MDH5MBPbIqV92AaeXatLxBI9gBaebbnrfifHhDYfgasaacH8akY=wiFfYdH8Gipec8Eeeu0xXdbba9frFj0=OqFfea0dXdd9vqai=hGuQ8kuc9pgc9s8qqaq=dirpe0xb9q8qiLsFr0=vr0=vr0dc8meaabaqaciaacaGaaeqabaqabeGadaaakeaacuWGYbGCgaWcaaaa@2E2B@) = (2*π*/*λ*)∫*dzn*(r→
 MathType@MTEF@5@5@+=feaafiart1ev1aaatCvAUfKttLearuWrP9MDH5MBPbIqV92AaeXatLxBI9gBaebbnrfifHhDYfgasaacH8akY=wiFfYdH8Gipec8Eeeu0xXdbba9frFj0=OqFfea0dXdd9vqai=hGuQ8kuc9pgc9s8qqaq=dirpe0xb9q8qiLsFr0=vr0=vr0dc8meaabaqaciaacaGaaeqabaqabeGadaaakeaacuWGYbGCgaWcaaaa@2E2B@, *z*), where *n*(r→
 MathType@MTEF@5@5@+=feaafiart1ev1aaatCvAUfKttLearuWrP9MDH5MBPbIqV92AaeXatLxBI9gBaebbnrfifHhDYfgasaacH8akY=wiFfYdH8Gipec8Eeeu0xXdbba9frFj0=OqFfea0dXdd9vqai=hGuQ8kuc9pgc9s8qqaq=dirpe0xb9q8qiLsFr0=vr0=vr0dc8meaabaqaciaacaGaaeqabaqabeGadaaakeaacuWGYbGCgaWcaaaa@2E2B@, *z*) is the 3D distribution of refractive index. The amplitude distribution after the object is written as

*A*(r→
 MathType@MTEF@5@5@+=feaafiart1ev1aaatCvAUfKttLearuWrP9MDH5MBPbIqV92AaeXatLxBI9gBaebbnrfifHhDYfgasaacH8akY=wiFfYdH8Gipec8Eeeu0xXdbba9frFj0=OqFfea0dXdd9vqai=hGuQ8kuc9pgc9s8qqaq=dirpe0xb9q8qiLsFr0=vr0=vr0dc8meaabaqaciaacaGaaeqabaqabeGadaaakeaacuWGYbGCgaWcaaaa@2E2B@, *t*) = ∫*dzS*(r→
 MathType@MTEF@5@5@+=feaafiart1ev1aaatCvAUfKttLearuWrP9MDH5MBPbIqV92AaeXatLxBI9gBaebbnrfifHhDYfgasaacH8akY=wiFfYdH8Gipec8Eeeu0xXdbba9frFj0=OqFfea0dXdd9vqai=hGuQ8kuc9pgc9s8qqaq=dirpe0xb9q8qiLsFr0=vr0=vr0dc8meaabaqaciaacaGaaeqabaqabeGadaaakeaacuWGYbGCgaWcaaaa@2E2B@, *z*)*T*[r→
 MathType@MTEF@5@5@+=feaafiart1ev1aaatCvAUfKttLearuWrP9MDH5MBPbIqV92AaeXatLxBI9gBaebbnrfifHhDYfgasaacH8akY=wiFfYdH8Gipec8Eeeu0xXdbba9frFj0=OqFfea0dXdd9vqai=hGuQ8kuc9pgc9s8qqaq=dirpe0xb9q8qiLsFr0=vr0=vr0dc8meaabaqaciaacaGaaeqabaqabeGadaaakeaacuWGYbGCgaWcaaaa@2E2B@-r→
 MathType@MTEF@5@5@+=feaafiart1ev1aaatCvAUfKttLearuWrP9MDH5MBPbIqV92AaeXatLxBI9gBaebbnrfifHhDYfgasaacH8akY=wiFfYdH8Gipec8Eeeu0xXdbba9frFj0=OqFfea0dXdd9vqai=hGuQ8kuc9pgc9s8qqaq=dirpe0xb9q8qiLsFr0=vr0=vr0dc8meaabaqaciaacaGaaeqabaqabeGadaaakeaacuWGYbGCgaWcaaaa@2E2B@_*S*_(*t*), *z*],     (3)

where r→
 MathType@MTEF@5@5@+=feaafiart1ev1aaatCvAUfKttLearuWrP9MDH5MBPbIqV92AaeXatLxBI9gBaebbnrfifHhDYfgasaacH8akY=wiFfYdH8Gipec8Eeeu0xXdbba9frFj0=OqFfea0dXdd9vqai=hGuQ8kuc9pgc9s8qqaq=dirpe0xb9q8qiLsFr0=vr0=vr0dc8meaabaqaciaacaGaaeqabaqabeGadaaakeaacuWGYbGCgaWcaaaa@2E2B@_*S*_(*t*) is the instantaneous position of the 2D raster scan.

The incoherent imaging mode is obtained with a spatially integrating detector, leading to a temporal signal proportional to the integrated intensity, ∫*d*^2^*r*|*A*(r→
 MathType@MTEF@5@5@+=feaafiart1ev1aaatCvAUfKttLearuWrP9MDH5MBPbIqV92AaeXatLxBI9gBaebbnrfifHhDYfgasaacH8akY=wiFfYdH8Gipec8Eeeu0xXdbba9frFj0=OqFfea0dXdd9vqai=hGuQ8kuc9pgc9s8qqaq=dirpe0xb9q8qiLsFr0=vr0=vr0dc8meaabaqaciaacaGaaeqabaqabeGadaaakeaacuWGYbGCgaWcaaaa@2E2B@, *t*)|^2^, which is stored in the computer. The data corresponding to each hologram line is cut from the signal, and band pass filtered to extract the term oscillating at Ω. The lines are then rearranged in a 2D format. The resulting hologram amplitude is found to be

*H_I_*(r→
 MathType@MTEF@5@5@+=feaafiart1ev1aaatCvAUfKttLearuWrP9MDH5MBPbIqV92AaeXatLxBI9gBaebbnrfifHhDYfgasaacH8akY=wiFfYdH8Gipec8Eeeu0xXdbba9frFj0=OqFfea0dXdd9vqai=hGuQ8kuc9pgc9s8qqaq=dirpe0xb9q8qiLsFr0=vr0=vr0dc8meaabaqaciaacaGaaeqabaqabeGadaaakeaacuWGYbGCgaWcaaaa@2E2B@) = ∫*dzI*(r→
 MathType@MTEF@5@5@+=feaafiart1ev1aaatCvAUfKttLearuWrP9MDH5MBPbIqV92AaeXatLxBI9gBaebbnrfifHhDYfgasaacH8akY=wiFfYdH8Gipec8Eeeu0xXdbba9frFj0=OqFfea0dXdd9vqai=hGuQ8kuc9pgc9s8qqaq=dirpe0xb9q8qiLsFr0=vr0=vr0dc8meaabaqaciaacaGaaeqabaqabeGadaaakeaacuWGYbGCgaWcaaaa@2E2B@, *z*) ⊕ [*p*_1_(r→
 MathType@MTEF@5@5@+=feaafiart1ev1aaatCvAUfKttLearuWrP9MDH5MBPbIqV92AaeXatLxBI9gBaebbnrfifHhDYfgasaacH8akY=wiFfYdH8Gipec8Eeeu0xXdbba9frFj0=OqFfea0dXdd9vqai=hGuQ8kuc9pgc9s8qqaq=dirpe0xb9q8qiLsFr0=vr0=vr0dc8meaabaqaciaacaGaaeqabaqabeGadaaakeaacuWGYbGCgaWcaaaa@2E2B@, *z*)p*_2_(r→
 MathType@MTEF@5@5@+=feaafiart1ev1aaatCvAUfKttLearuWrP9MDH5MBPbIqV92AaeXatLxBI9gBaebbnrfifHhDYfgasaacH8akY=wiFfYdH8Gipec8Eeeu0xXdbba9frFj0=OqFfea0dXdd9vqai=hGuQ8kuc9pgc9s8qqaq=dirpe0xb9q8qiLsFr0=vr0=vr0dc8meaabaqaciaacaGaaeqabaqabeGadaaakeaacuWGYbGCgaWcaaaa@2E2B@, *z*)],     (4)

where ⊕ symbolizes a convolution integral, *I*(r→
 MathType@MTEF@5@5@+=feaafiart1ev1aaatCvAUfKttLearuWrP9MDH5MBPbIqV92AaeXatLxBI9gBaebbnrfifHhDYfgasaacH8akY=wiFfYdH8Gipec8Eeeu0xXdbba9frFj0=OqFfea0dXdd9vqai=hGuQ8kuc9pgc9s8qqaq=dirpe0xb9q8qiLsFr0=vr0=vr0dc8meaabaqaciaacaGaaeqabaqabeGadaaakeaacuWGYbGCgaWcaaaa@2E2B@, *z*) = |*T*(r→
 MathType@MTEF@5@5@+=feaafiart1ev1aaatCvAUfKttLearuWrP9MDH5MBPbIqV92AaeXatLxBI9gBaebbnrfifHhDYfgasaacH8akY=wiFfYdH8Gipec8Eeeu0xXdbba9frFj0=OqFfea0dXdd9vqai=hGuQ8kuc9pgc9s8qqaq=dirpe0xb9q8qiLsFr0=vr0=vr0dc8meaabaqaciaacaGaaeqabaqabeGadaaakeaacuWGYbGCgaWcaaaa@2E2B@, *z*)|^2^, and *p*_1,2_(r→
 MathType@MTEF@5@5@+=feaafiart1ev1aaatCvAUfKttLearuWrP9MDH5MBPbIqV92AaeXatLxBI9gBaebbnrfifHhDYfgasaacH8akY=wiFfYdH8Gipec8Eeeu0xXdbba9frFj0=OqFfea0dXdd9vqai=hGuQ8kuc9pgc9s8qqaq=dirpe0xb9q8qiLsFr0=vr0=vr0dc8meaabaqaciaacaGaaeqabaqabeGadaaakeaacuWGYbGCgaWcaaaa@2E2B@, *z*) are the inverse Fourier transforms of the defocused pupil distributions (eq.1b). The superscript * stands for complex conjugate. The hologram is thus the convolution of the object intensity with a spherical wave, i.e. an in-line single-sideband Gabor hologram. In Fourier space, the hologram can be written as

H˜I(ρ→)=∫dzI˜(ρ→;z)[P˜1(ρ→;z)⊗P˜2(ρ→;z)],     (5)
 MathType@MTEF@5@5@+=feaafiart1ev1aaatCvAUfKttLearuWrP9MDH5MBPbIqV92AaeXatLxBI9gBaebbnrfifHhDYfgasaacH8akY=wiFfYdH8Gipec8Eeeu0xXdbba9frFj0=OqFfea0dXdd9vqai=hGuQ8kuc9pgc9s8qqaq=dirpe0xb9q8qiLsFr0=vr0=vr0dc8meaabaqaciaacaGaaeqabaqabeGadaaakeaacuWGibasgaacamaaBaaaleaacqWGjbqsaeqaaOGaeiikaGccciGaf8xWdiNbaSaacqGGPaqkcqGH9aqpdaWdbaqaaiabdsgaKjabdQha6jqbdMeajzaaiaaaleqabeqdcqGHRiI8aOGaeiikaGIaf8xWdiNbaSaacqGG7aWocqWG6bGEcqGGPaqkdaWadaqaaiqbdcfaqzaaiaWaaSbaaSqaaiabigdaXaqabaGccqGGOaakcuWFbpGCgaWcaiabcUda7iabdQha6jabcMcaPiabgEPielqbdcfaqzaaiaWaaSbaaSqaaiabikdaYaqabaGccqGGOaakcuWFbpGCgaWcaiabcUda7iabdQha6jabcMcaPaGaay5waiaaw2faaiabcYcaSiaaxMaacaWLjaWaaeWaaeaacqaI1aqnaiaawIcacaGLPaaaaaa@58FF@

where ⊕ symbolizes a correlation integral. With the pupils of eq.2, we find, in the paraxial approximation,

H˜I(ρ→)=∫dzI˜(ρ→;z)exp⁡[iπλ(z0+z)ρ2]circ(ρ/ρMAX),     (6)
 MathType@MTEF@5@5@+=feaafiart1ev1aaatCvAUfKttLearuWrP9MDH5MBPbIqV92AaeXatLxBI9gBaebbnrfifHhDYfgasaacH8akY=wiFfYdH8Gipec8Eeeu0xXdbba9frFj0=OqFfea0dXdd9vqai=hGuQ8kuc9pgc9s8qqaq=dirpe0xb9q8qiLsFr0=vr0=vr0dc8meaabaqaciaacaGaaeqabaqabeGadaaakeaacuWGibasgaacamaaBaaaleaacqWGjbqsaeqaaOGaeiikaGccciGaf8xWdiNbaSaacqGGPaqkcqGH9aqpdaWdbaqaaiabdsgaKjabdQha6jqbdMeajzaaiaaaleqabeqdcqGHRiI8aOGaeiikaGIaf8xWdiNbaSaacqGG7aWocqWG6bGEcqGGPaqkcyGGLbqzcqGG4baEcqGGWbaCdaWadaqaaiabdMgaPjab=b8aWjab=T7aSjabcIcaOiabdQha6naaBaaaleaacqaIWaamaeqaaOGaey4kaSIaemOEaONaeiykaKIae8xWdi3aaWbaaSqabeaacqaIYaGmaaaakiaawUfacaGLDbaacqWGJbWycqWGPbqAcqWGYbGCcqWGJbWycqGGOaakcqWFbpGCcqGGVaWlcqWFbpGCdaWgaaWcbaGaemyta0KaemyqaeKaemiwaGfabeaakiabcMcaPiabcYcaSiaaxMaacaWLjaWaaeWaaeaacqaI2aGnaiaawIcacaGLPaaaaaa@67E3@

which is the Fourier transform of the Fresnel hologram of the object's intensity distribution.

The coherent imaging mode is obtained by using a pinhole or a point detector at the center of a conjugate plane of the pupil of the objective. This leads to the hologram amplitude

*H_C_*(r→
 MathType@MTEF@5@5@+=feaafiart1ev1aaatCvAUfKttLearuWrP9MDH5MBPbIqV92AaeXatLxBI9gBaebbnrfifHhDYfgasaacH8akY=wiFfYdH8Gipec8Eeeu0xXdbba9frFj0=OqFfea0dXdd9vqai=hGuQ8kuc9pgc9s8qqaq=dirpe0xb9q8qiLsFr0=vr0=vr0dc8meaabaqaciaacaGaaeqabaqabeGadaaakeaacuWGYbGCgaWcaaaa@2E2B@) = ∫*dz*[*T*(r→
 MathType@MTEF@5@5@+=feaafiart1ev1aaatCvAUfKttLearuWrP9MDH5MBPbIqV92AaeXatLxBI9gBaebbnrfifHhDYfgasaacH8akY=wiFfYdH8Gipec8Eeeu0xXdbba9frFj0=OqFfea0dXdd9vqai=hGuQ8kuc9pgc9s8qqaq=dirpe0xb9q8qiLsFr0=vr0=vr0dc8meaabaqaciaacaGaaeqabaqabeGadaaakeaacuWGYbGCgaWcaaaa@2E2B@, *z*) ⊕ *p*_1_(r→
 MathType@MTEF@5@5@+=feaafiart1ev1aaatCvAUfKttLearuWrP9MDH5MBPbIqV92AaeXatLxBI9gBaebbnrfifHhDYfgasaacH8akY=wiFfYdH8Gipec8Eeeu0xXdbba9frFj0=OqFfea0dXdd9vqai=hGuQ8kuc9pgc9s8qqaq=dirpe0xb9q8qiLsFr0=vr0=vr0dc8meaabaqaciaacaGaaeqabaqabeGadaaakeaacuWGYbGCgaWcaaaa@2E2B@, *z*)][*T*(r→
 MathType@MTEF@5@5@+=feaafiart1ev1aaatCvAUfKttLearuWrP9MDH5MBPbIqV92AaeXatLxBI9gBaebbnrfifHhDYfgasaacH8akY=wiFfYdH8Gipec8Eeeu0xXdbba9frFj0=OqFfea0dXdd9vqai=hGuQ8kuc9pgc9s8qqaq=dirpe0xb9q8qiLsFr0=vr0=vr0dc8meaabaqaciaacaGaaeqabaqabeGadaaakeaacuWGYbGCgaWcaaaa@2E2B@, *z*) ⊕ *p*_2_(r→
 MathType@MTEF@5@5@+=feaafiart1ev1aaatCvAUfKttLearuWrP9MDH5MBPbIqV92AaeXatLxBI9gBaebbnrfifHhDYfgasaacH8akY=wiFfYdH8Gipec8Eeeu0xXdbba9frFj0=OqFfea0dXdd9vqai=hGuQ8kuc9pgc9s8qqaq=dirpe0xb9q8qiLsFr0=vr0=vr0dc8meaabaqaciaacaGaaeqabaqabeGadaaakeaacuWGYbGCgaWcaaaa@2E2B@, *z*)]*.     (7)

Where again, ⊕ symbolizes a convolution product, and the superscript * stands for complex conjugate. In Fourier space,

H˜C(ρ→)=∫dzT˜(ρ→;z)P˜1(ρ→;z)⊗T˜(ρ→;z)P˜2(ρ→;z).     (8)
 MathType@MTEF@5@5@+=feaafiart1ev1aaatCvAUfKttLearuWrP9MDH5MBPbIqV92AaeXatLxBI9gBaebbnrfifHhDYfgasaacH8akY=wiFfYdH8Gipec8Eeeu0xXdbba9frFj0=OqFfea0dXdd9vqai=hGuQ8kuc9pgc9s8qqaq=dirpe0xb9q8qiLsFr0=vr0=vr0dc8meaabaqaciaacaGaaeqabaqabeGadaaakeaacuWGibasgaacamaaBaaaleaacqWGdbWqaeqaaOGaeiikaGccciGaf8xWdiNbaSaacqGGPaqkcqGH9aqpdaWdbaqaaiabdsgaKjabdQha6jqbdsfauzaaiaaaleqabeqdcqGHRiI8aOGaeiikaGIaf8xWdiNbaSaacqGG7aWocqWG6bGEcqGGPaqkcuWGqbaugaacamaaBaaaleaacqaIXaqmaeqaaOGaeiikaGIaf8xWdiNbaSaacqGG7aWocqWG6bGEcqGGPaqkcqGHxkcXcuWGubavgaacaiabcIcaOiqb=f8aYzaalaGaei4oaSJaemOEaONaeiykaKIafmiuaaLbaGaadaWgaaWcbaGaeGOmaidabeaakiabcIcaOiqb=f8aYzaalaGaei4oaSJaemOEaONaeiykaKIaeiOla4IaaCzcaiaaxMaadaqadaqaaiabiIda4aGaayjkaiaawMcaaaaa@5E5B@

With the pupils of eq.2, we find

H˜C(ρ→)=∫dzT˜∗(0;z)T˜(ρ→;z)exp⁡[iπλ(z0+z)ρ2]circ(ρ/ρMAX).     (9)
 MathType@MTEF@5@5@+=feaafiart1ev1aaatCvAUfKttLearuWrP9MDH5MBPbIqV92AaeXatLxBI9gBaebbnrfifHhDYfgasaacH8akY=wiFfYdH8Gipec8Eeeu0xXdbba9frFj0=OqFfea0dXdd9vqai=hGuQ8kuc9pgc9s8qqaq=dirpe0xb9q8qiLsFr0=vr0=vr0dc8meaabaqaciaacaGaaeqabaqabeGadaaakeaacuWGibasgaacamaaBaaaleaacqWGdbWqaeqaaOGaeiikaGccciGaf8xWdiNbaSaacqGGPaqkcqGH9aqpdaWdbaqaaiabdsgaKjabdQha6jqbdsfauzaaiaGaey4fIOIaeiikaGIaeGimaaJaei4oaSJaemOEaONaeiykaKIafmivaqLbaGaaaSqabeqaniabgUIiYdGccqGGOaakcuWFbpGCgaWcaiabcUda7iabdQha6jabcMcaPiGbcwgaLjabcIha4jabcchaWnaadmaabaGaemyAaKMae8hWdaNae83UdWMaeiikaGIaemOEaO3aaSbaaSqaaiabicdaWaqabaGccqGHRaWkcqWG6bGEcqGGPaqkcqWFbpGCdaahaaWcbeqaaiabikdaYaaaaOGaay5waiaaw2faaiabdogaJjabdMgaPjabdkhaYjabdogaJjabcIcaOiab=f8aYjabc+caViab=f8aYnaaBaaaleaacqWGnbqtcqWGbbqqcqWGybawaeqaaOGaeiykaKIaeiOla4IaaCzcaiaaxMaadaqadaqaaiabiMda5aGaayjkaiaawMcaaaaa@6F41@

Aside from an inconsequential complex constant (the first term under the integral), eq.9 is the Fourier transform of the Fresnel hologram of the object's complex amplitude distribution. Thus, the reconstruction of the hologram recorded in the coherent mode carries a quantitative measure of the object's phase distribution.

## 3- Experimental holographic microscope

The experimental system sketched in fig. 1 uses a 20X objective with NA = 0.42 (Mitutoyo Plan Apo). The detectors are standard photomultiplier tubes (Hamamatsu). In the coherent mode, the signal is measured through a 10 *μm *pinhole placed in a conjugate plane of the objective's pupil. The specimen is mounted on a 2D piezo scanning stage (Physik Instrumente). The area scanned is 150 × 150 *μm*^2 ^divided in 2000 lines with 75 nm line spacing. Each line is sampled at 10,000 samples per line. The data acquisition system is a Gage Scope with a sampling rate set at 10^5 ^samples per sec. The Fresnel pattern projected on the object has a diameter *a *~ 50 *μm*, and a Fresnel number ~16. At the wavelength of 532 nm, this corresponds to a radius of curvature of the spherical wave *z*_0 _~ 75 *μm *in the focal plane of the objective, and an effective numerical aperture *NA *= sin*α *= *a*/*z*_0 _~ 0.34. The Rayleigh transverse resolution limit is thus *λ*/2*NA *~ 0.8 *μm*, a number which has been verified in previous experiments [7].

The high resolution sampling is needed for two reasons: to insure an adequate representation of the hologram phase, and to satisfy the Shannon/Nyquist sampling theorem in the signal demodulation. The sampling theorem requires a minimum of two samples per resolution element. Thus, a single intensity image with size 150 × 150 *μm*^2^, and 0.8 *μm *resolution is adequately represented by an array of 375 × 375 samples. To represent a complex hologram of the same size, however, a finer sampling is needed to capture the hologram phase. The width of the outermost Fresnel zone of the pattern scanning the specimen is equal to the resolution limit (0.8 *μm*), and represents a phase excursion of *π*. We found experimentally that more than five samples per resolution element were needed to capture this phase excursion with sufficient resolution. In the present experiment, we chose a sampling interval of 75 nm, corresponding to ~10 samples per resolution element, and a phase sampling interval ~*π*/10. Note that in standard digital holography, a high resolution pixellated detector (e. g. CCD array) is needed to capture the hologram phase via interferences in the spatial domain. In this case, the detector's spatial resolution is often a factor limiting the resolution. In contrast, the scanning holographic method captures the hologram phase via the temporal modulation of the scanning pattern integrated on a non-imaging detector. The resolution of digital holography is limited by the spatial sampling of the detector, while that of scanning holography is limited by the temporal sampling rate.

From the Shannon/Nyquist sampling theorem, the signal modulation frequency must be smaller than 1/4 the sampling rate, and the band pass of the filter demodulating the signal must be smaller than the modulation frequency. In our set up, the sampling rate is 10^5 ^*Hz*, the modulation frequency is 25 *kHz*, obtained with a ramp-driven electro-optic phase modulator (Linos), and the filter band pass is 20 *kHz*. Note that this demodulation process is done digitally in the computer, since the scanning effects a mapping from time to space, temporal frequencies are directly related to sample numbers. In the spatial domain, each line of the hologram is chosen to have 10,000 samples, corresponding to the sampling rate of 10^5 ^*Hz*. The Fourier transform of each line, which also has 10,000 samples, has two modulation bands centered at sample 2500, and 7500 (corresponding to the two frequency sidebands at ± 25 *kHz*). After adjusting the initial phase of each line to that of a reference signal (see fig. 1), one of the modulation band is extracted to form a hologram line of 2000 samples (corresponding to the band width of 20 *kHz*). The final hologram is then assembled in a 2D array of 2000 × 2000 samples. The hologram is reconstructed by standard Fresnel back-propagation using a Matlab code (see ref. 6, 7 for more details). In the present experiment, the acquisition time of one hologram is limited by the speed of the scanning stage to about 4 min. With a Pentium D-820 dual processor, it takes less than 10s to construct the 2D hologram, and less than 50s to reconstruct a stack of 10 axial images.

## 4- Experimental results

**Figure 2:**
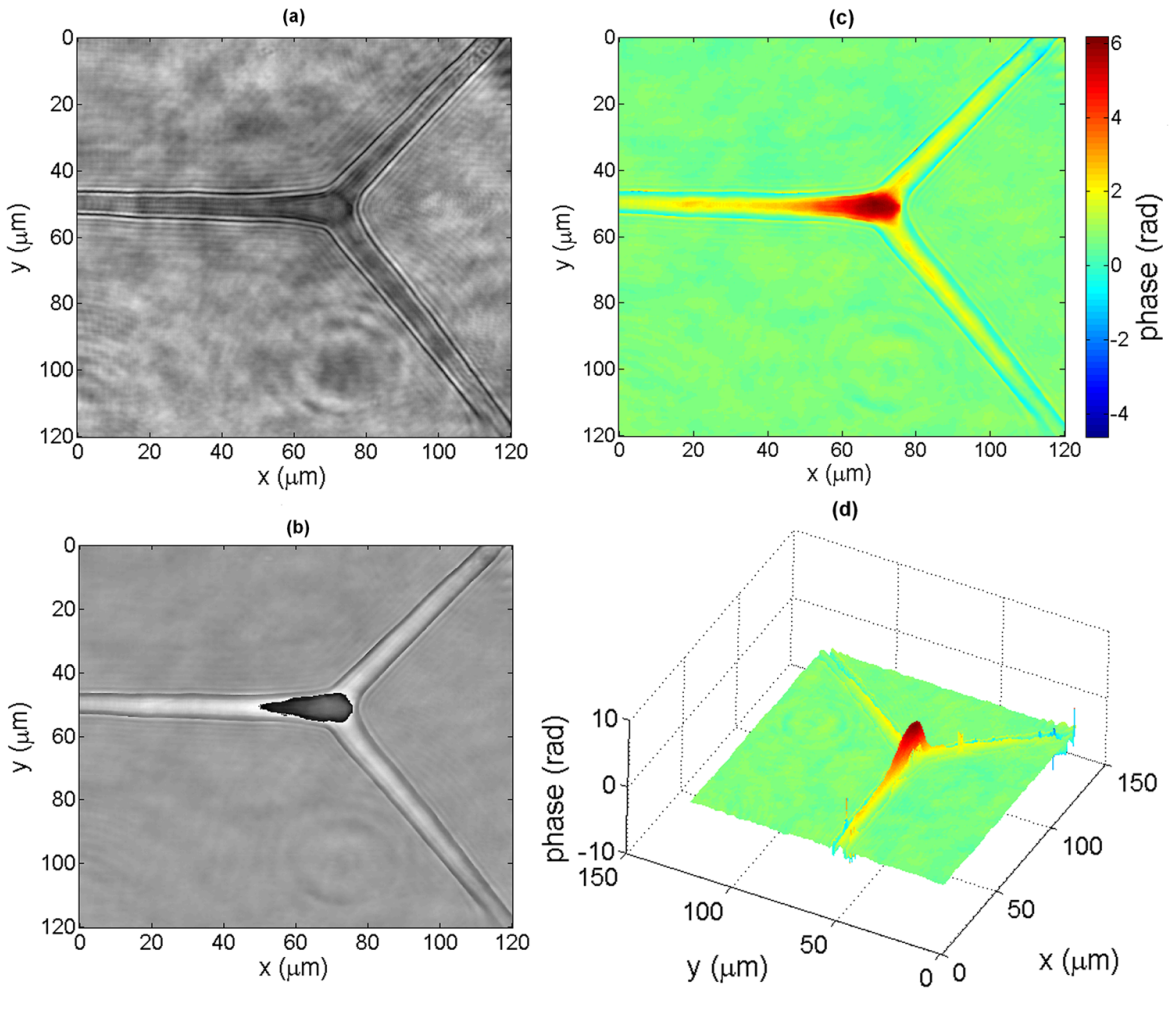
**Holographic reconstruction of a spongila spicule**. Reconstruction of a hologram recorded in coherent mode. The object is a siliceous three-pronged spongilla spicule. (a) absolute value of the reconstruction amplitude, (b) wrapped phase map of the optical thickness of the object relative to the mounting medium (black = -*π*/2 + *n*2*π *; gray = *n*2*π *; white = *π*/2 + *n*2*π*), (c) unwrapped phase map (the color code indicates the phase in radians), (d) 3D phase profile of the object.

**Figure 3:**
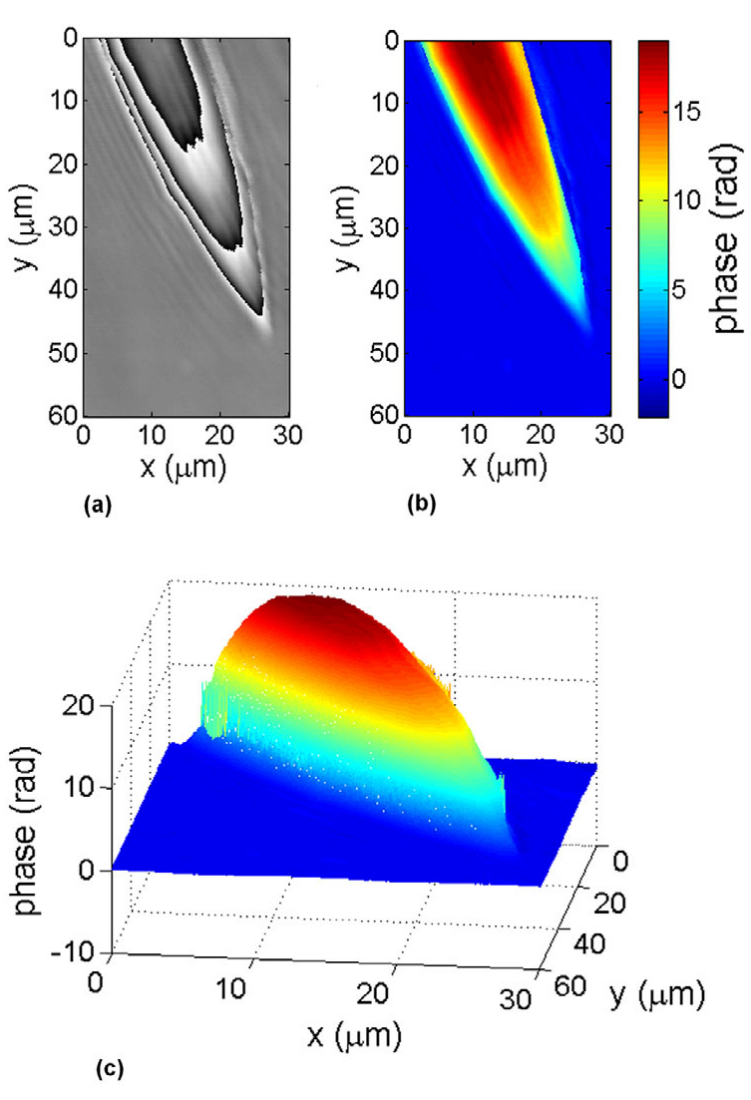
**Detail of the reconstruction of a spongila spicule**. Details of the phase profile of the tip of a thicker spicule. (a) wrapped phase map (black = -*π*/2 + *n*2*π *; gray = *n*2*π *; white = *π*/2 + *n*2*π*), (b) unwrapped phase map (the color code indicates the phase in radians), (c) 3D phase profile.

Fig. 2 shows the amplitude's absolute value (a) and the phase (b) of the reconstruction of a typical three-pronged siliceous spicule forming the exoskeleton of a marine sponge (genus spongilla) (slide from Carolina supply). This object is almost a pure phase object, but the edges are rendered visible in the amplitude image due to diffraction. Fig. 2c is the unwrapped phase map, using the SPUA2 software from Phasevision. The 3D profile of this object is shown in fig. 2d. The phase provides a measure of the optical thickness of the object relative to the mounting medium. Fig. 3 shows the details of the tip of a thicker spicule.

**Figure 4:**
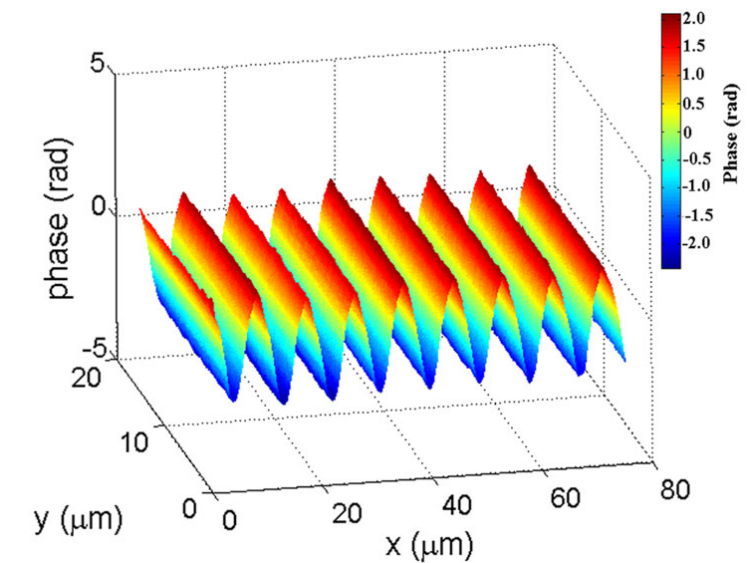
**Holographic reconstruction of the phase profile of a triangular transmission phase grating**. Quantitative phase profile of a 110 l/mm relief phase grating with a 7° triangular profile embossed in transparent epoxy. The average modulation depth of the profile is measured to be ≈ 570 ± 5*nm*, using a refractive index *n *= 1.54 for the epoxy.

**Figure 5:**
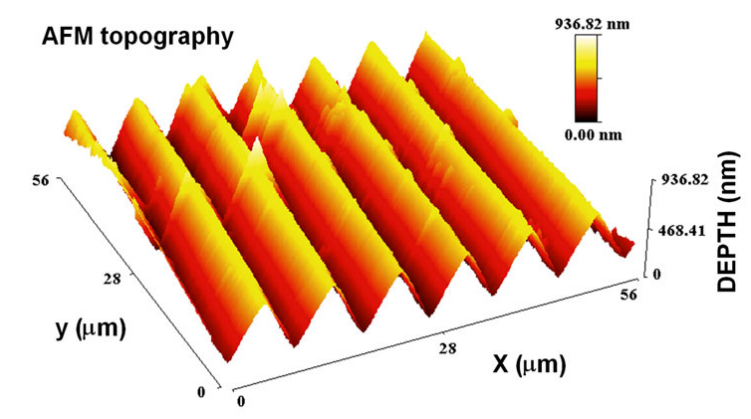
**AFM topographic profile of the phase grating**. Atomic force microscope topographic profile of a different section of the same grating. The average modulation depth is measured to be ≈ 540 ± 10*nm*.

**Figure 6:**
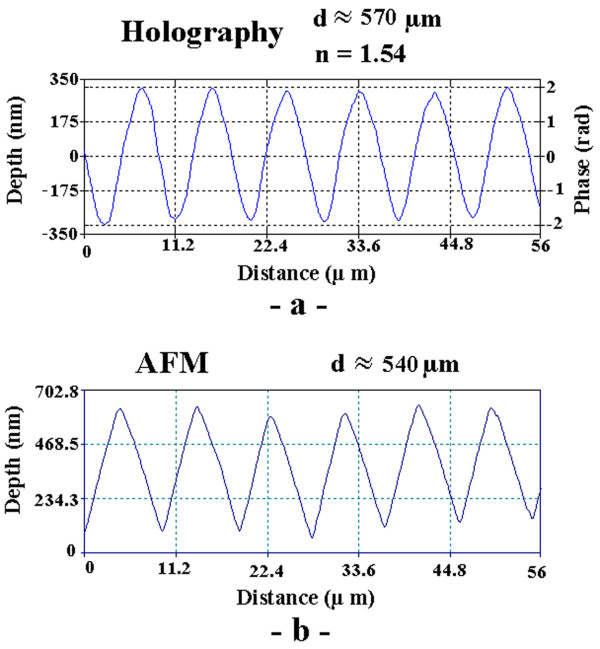
**Comparison of the grating profile obtained from holography, and from AFM**. (a) Depth profile of a small section of the grating obtained from the holographic data using *n *= 1.54 for the epoxy. (b) Depth profile of another grating section obtained from the AFM data. The differences are discussed in the text.

To test the quantitative value of these results, we recorded the hologram of a relief phase grating with 110 lines per mm embossed in a transparent epoxy (Edmund Scientific). The phase profile of a section of the grating, reconstructed from the hologram, is shown in fig. 4. The absolute value of the reconstruction is nearly uniform since this object is almost a pure phase object. The average peak-to-peak phase modulation is measured to be Φ = 3.75 ± 0.025 *rad*. Using the refractive index of the epoxy *n *= 1.54, given by the manufacturer, this corresponds to a peak to peak profile modulation *d *= *λ*Φ/2*π*(*n *- 1) ≈ 570 ± 5 *nm*, for a wavelength of 532 nm. For comparison, fig. 5 shows the topography of a small part of the grating obtained with an atomic force microscope (AFM) in the non-contact mode. The average modulation depth is found to be ≈ 540 ± 10 *nm*. The larger standard deviation is due to the smaller sample size of the AFM data. The difference between these two results, which amount to less than 10% is not too surprising considering the very different principles of the two measurement methods. This difference could be due, for example, to an underestimation of the refractive index of the epoxy, a calibration error of the AFM, or the unlikely, but possible fact that the AFM probe did not reach the very bottom of the grooves. Most likely however, the difference is due to the fact that the limited spatial resolution of the holographic imaging method acts as a low pass filter, as it does in any imaging method. The AFM point-like method does not have this limitation. This effect is further illustrated in fig. 6, which shows the line profiles of a ~50 *μm *long trace across the grating obtained from the holographic data (fig. 6a), and from the AFM data (fig. 6b). This figure reveals that the AFM data is close to the expected 7° saw-tooth profile stipulated by the manufacturer, while the profile obtained with the holographic method appears to be less sharply triangular. The representation of a band-limited phase function *f*(r→
 MathType@MTEF@5@5@+=feaafiart1ev1aaatCvAUfKttLearuWrP9MDH5MBPbIqV92AaeXatLxBI9gBaebbnrfifHhDYfgasaacH8akY=wiFfYdH8Gipec8Eeeu0xXdbba9frFj0=OqFfea0dXdd9vqai=hGuQ8kuc9pgc9s8qqaq=dirpe0xb9q8qiLsFr0=vr0=vr0dc8meaabaqaciaacaGaaeqabaqabeGadaaakeaacuWGYbGCgaWcaaaa@2E2B@) = exp[*i*Φ(r→
 MathType@MTEF@5@5@+=feaafiart1ev1aaatCvAUfKttLearuWrP9MDH5MBPbIqV92AaeXatLxBI9gBaebbnrfifHhDYfgasaacH8akY=wiFfYdH8Gipec8Eeeu0xXdbba9frFj0=OqFfea0dXdd9vqai=hGuQ8kuc9pgc9s8qqaq=dirpe0xb9q8qiLsFr0=vr0=vr0dc8meaabaqaciaacaGaaeqabaqabeGadaaakeaacuWGYbGCgaWcaaaa@2E2B@)] is limited by the Bernstein theorem [12] in such a way that the gradient of its phase is bounded by |∇→
 MathType@MTEF@5@5@+=feaafiart1ev1aaatCvAUfKttLearuWrP9MDH5MBPbIqV92AaeXatLxBI9gBaebbnrfifHhDYfgasaacH8akY=wiFfYdH8Gipec8Eeeu0xXdbba9frFj0=OqFfea0dXdd9vqai=hGuQ8kuc9pgc9s8qqaq=dirpe0xb9q8qiLsFr0=vr0=vr0dc8meaabaqaciaacaGaaeqabaqabeGadaaakeaacuGHhis0gaWcaaaa@2E44@Φ(r→
 MathType@MTEF@5@5@+=feaafiart1ev1aaatCvAUfKttLearuWrP9MDH5MBPbIqV92AaeXatLxBI9gBaebbnrfifHhDYfgasaacH8akY=wiFfYdH8Gipec8Eeeu0xXdbba9frFj0=OqFfea0dXdd9vqai=hGuQ8kuc9pgc9s8qqaq=dirpe0xb9q8qiLsFr0=vr0=vr0dc8meaabaqaciaacaGaaeqabaqabeGadaaakeaacuWGYbGCgaWcaaaa@2E2B@)| ≤ 2*πρ*_max_, where *ρ*_max _= *NA*/*λ *is the cut-off frequency of the objective. This limit corresponds physically to a maximum allowed phase excursion of *π *per resolution element. In our case, *ρ*_max _~ 0.4 *μm*^-1^, and the slope of the triangular phase profile changes from + 0.75 *μm*^-1 ^to -0.75 *μm*^-1 ^at the apex of the triangle. In these conditions, the sharp tip of the triangular phase profile is expected to be smoothed out by the limited spatial resolution. This of course is not the case with the point-like AFM method. In principle, this effect could be corrected by post-processing, and deconvolution of the images, using the knowledge, or at least an estimate model, of the system's transfer function. It is interesting to note that the holographic data was recorded in ~4 min. (an acquisition time that could be reduced with a faster scanning device), while the capture of the AFM data took more than 4 hrs.

## 5-Summary

Quantitative phase imaging of unstained biological specimens and relief optical surfaces using scanning holographic microscopy has been demonstrated experimentally for the first time. Quantitative phase measurement of biological specimens was first demonstrated with digital holographic microscopy [2], which is a coherent imaging process. The advantage of the scanning holographic method used in this work is that it is possible to obtain holograms in both the coherent and the incoherent imaging modes simultaneously. This opens up new possibilities for multimodal imaging, making it possible, for example, to acquire absorption images, fluorescence images, and quantitative phase images of three-dimensional specimens simultaneously using the same scanning holographic microscope.

## Competing interests

The author(s) declare that they have no competing interests.

## Authors' contributions

G. I. carried the experiment, collected the data, and wrote the manuscript. Y. T. wrote the MatLab codes to reconstruct the holograms. J. L. applied the phase unwrapping algorithm to the reconstructed images, and reviewed the manuscript.
